# The predictive value of PSMA PET/CT in determining pathological upgrading of prostate cancer: A pooling up analysis

**DOI:** 10.3389/fonc.2025.1525890

**Published:** 2025-09-15

**Authors:** Yue Liu, Shu-Pei Qu, Ling-Yun Zhai

**Affiliations:** ^1^ Department of Nursing, The First Affiliated Hospital of Chongqing Medical University, Chongqing, China; ^2^ Department of Gastrointestinal Surgery, The First Affiliated Hospital of Chongqing Medical University, Chongqing, China; ^3^ Department of Urology, The Second Affiliated Hospital of Chongqing Medical University, Chongqing, China

**Keywords:** prostate cancer, radical prostatectomy, upgrading, PSMA PET/CT, predictive value

## Abstract

**Purpose:**

The issue of pathological upgrading following radical prostatectomy poses a significant challenge for urologists, and prostate-specific membrane antigen (PSMA) positron emission tomography-computed tomography(PET/CT) has gained increasing prominence as a preoperative assessment tool for patients with prostate cancer in recent years. This study aims to assess the diagnostic accuracy of PSMA PET/CT in predicting pathological upgrading after radical prostatectomy.

**Methods:**

We conducted a meta-analysis of diagnostic studies using data from the Cochrane CENTRAL, PubMed, Embase, Scopus, and Web of Science databases through March 2024. We strictly adhered to the guidelines outlined in the PRISMA statement for conducting this diagnostic meta-analysis. We computed the pooled diagnostic accuracy and evaluated heterogeneity while exploring potential sources of heterogeneity through subgroup analysis.

**Results:**

A total of 7 studies involving 507 patients were included in the final analysis. All participants had biopsy-confirmed prostate cancer and underwent radical prostatectomy. Prior to surgery, all patients underwent PSMA PET/CT imaging. The pooled diagnostic accuracy yielded a sensitivity of 0.68 (95% CI, 0.60 - 0.76) and specificity of 0.74 (95% CI, 0.59 - 0.85). The area under the summary receiver operating characteristic curve was calculated as 0.74 (95%CI, 0.70 - 0.78). Although heterogeneity was observed, its source remained unclear.

**Conclusion:**

The PSMA PET/CT demonstrates a moderate level of accuracy in predicting pathological upgrading following radical prostatectomy, making it a tool with potential clinical application value, particularly in the field of radiotherapy. However, further studies are warranted to enhance its relevance and applicability.

**Systematic Review Registration:**

https://www.crd.york.ac.uk/prospero/, identifier CRD42024503406.

## Introduction

Prostate cancer ranks fourth in terms of global incidence and is the second leading cause of cancer-related mortality among men in the United States (1). Optimal treatment selection for prostate cancer patients relies on disease risk stratification, with the widely used D’Amico system categorizing prostate cancer into high risk, moderate risk, low risk, and very low risk based on NCCN guidelines (2). Among various risk stratification systems, the Gleason score plays a pivotal role; however, previous studies have indicated potential inconsistencies between postoperative pathological Gleason scores and biopsy Gleason scores in patients who have undergone radical prostatectomy (3). This discrepancy can lead to preoperative misclassification and impact treatment decisions such as lymph node dissection or preservation of sexual nerves (4, 5). Particularly concerning active surveillance, which considers both patient quality of life and timely treatment opportunities, upgrading of the Gleason score may result in mistakenly enrolling patients who should have received radical treatment (6).

In previous studies, various methods have been explored to predict pathological upgrading in prostate cancer patients (7). Notably, multi-parametric magnetic resonance imaging has played a significant role in this aspect (8). Recently, PSMA PET/CT has gained widespread usage for diagnosing and staging prostate cancer (9); however, limited research exists on its potential for predicting pathological upgrading. Therefore, this study conducted a diagnostic meta-analysis to assess the diagnostic accuracy of PSMA PET/CT in predicting pathological upgrading of prostate cancer.

## Methods

### Literature search and selection criteria

The present meta-analysis was prospectively registered in PROSPERO (CRD42024503406) using the following search strategies: (upgrade or upgrading or upgraded or active surveillance [MESH]) and (PSMA OR prostate-specific membrane antigen). We conducted a comprehensive literature search of Cochrane CENTRAL, PubMed, Embase, Scopus, and Web of Science from their inception until March 2024. Only articles written in English were eligible for inclusion.

Inclusion criteria for the literature review were as follows: 1) Patients diagnosed with prostate cancer through biopsy and undergoing radical prostatectomy; 2) Patients who underwent PSMA PET/CT examination prior to radical prostatectomy; 3) Postoperative pathology of prostate cancer served as the gold standard; 4) Relevant data could be extracted. Exclusion criteria for the literature review included: 1) Studies with highly overlapping populations; 2) Reviews or meta-analyses; 3) Abstract-only papers, conference proceedings, or books.

### Screening strategies and data collection

After conducting the database search, two authors independently reviewed all titles and abstracts. If either author deemed an article eligible, both authors would thoroughly examine the entire article to determine its inclusion in the study. Subsequently, each author extracted relevant data and information from every included article. In case of discordant opinions between the two authors, they initially attempted to resolve the disagreement through consultation. If consensus could not be reached via consultation, third-party arbitration was sought for dispute resolution.

### Quality assessment and bias analysis

The Diagnostic Accuracy Research Quality Assessment (QUADAS - 2) (10) scale was used to evaluate the quality of the included literature, and Deeks’ funnel plot (11) was used to identify potential publication bias.

### Statistical analysis

We conducted a diagnostic meta-analysis in strict accordance with the PRISMA statement (12). Tables were generated to present true positives, false positives, true negatives, and false negatives. Sensitivity, specificity, positive likelihood ratio, negative likelihood ratio, diagnostic odds ratio (DOR), and 95% confidence intervals (CI) were calculated for each study. The data was combined and visualized through summary receiver operating characteristic (SROC) curve analysis and forest plot analysis. Heterogeneity was assessed using the I^2^ method (13) and potential sources of heterogeneity were explored through subgroup analysis. A bivariable mixed-effects regression model was employed for statistical analysis (14). Statistical significance was defined as P<0.05. Rev Man 5.3 and STATA 14’s MIDAS module were used for all statistical analyses (15).

## Results

### Eligible studies and quality assessment

**Figure 1:**
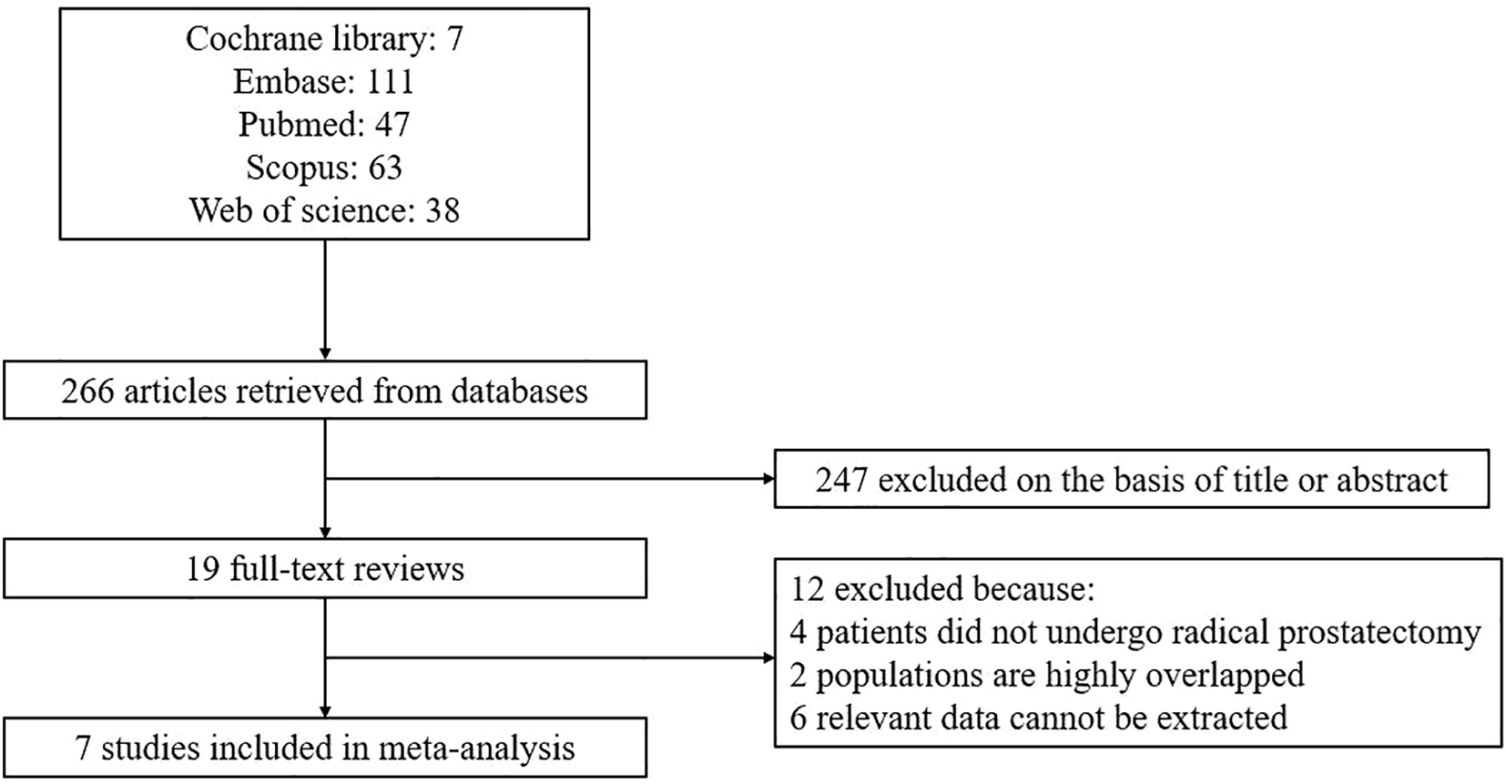
Study selection.

After conducting an initial literature search and eliminating duplicate articles, a total of 266 articles were identified. Following a thorough review of the titles and abstracts, 247 articles were excluded, leaving 19 articles for full-text assessment. Ultimately, this meta-analysis incorporated 7 studies (16–22). The process of article selection and the reasons for exclusion are visually presented in **Figure 1**.

The seven studies included in this analysis were all retrospective in nature, with one study being multi-center and the remaining six being single-center. In total, 507 patients diagnosed with prostate cancer through biopsy and subsequent radical prostatectomy were included across all studies. Prior to surgery, PSMA-PET examinations were conducted on all patients; specifically, six studies utilized 68Ga-PSMA-PET/CT while the remaining study used 18F-PSMA-PET/CT. It is worth noting that two studies enrolled patients with ISUP GG 1 biopsy results, one study enrolled those with ISUP GG 2 biopsy results, and four studies enrolled those with ISUP GG1 – 4 biopsy results. The term “upgrade” is defined as the presence of a higher pathological ISUP GG grade in radical prostatectomy specimens compared to the needle biopsy ISUP GG grade. Regarding the biopsy strategy, three studies employed a combination of systematic and MRI-targeted biopsies, while three studies solely relied on systematic biopsies. In terms of determining positive results from PSMA-PET/CT scans, one study utilized the primary score as the standard, another study employed a deep learning model, and five other studies used SUVmax-related indicators. **Table 1** provides an overview of the key characteristics found in the included literature.

**Table 1:** Summary of included studies.

First author, year	Number of patients	Patient characteristics	Biopsy process	Imaging modality	Positive test criteria	Reference standard	Positive result criteria
Akcay 2023 (16)	73	Biopsy ISUP GG 1	NA	68Ga-PSMA-11 PET/CT	PRIMARY score 3-5	RP pathology	RP ISUP GG ≥ 2
Esen 2024 (17)	41	Biopsy ISUP GG 1	systematic and MRI‐targeted biopsies	68Ga‐PSMA‐11 PET/CT	SUVmax ≥ 5.6	RP pathology	RP ISUP GG ≥ 2
Hu 2022 (18)	62	Biopsy ISUP GG 1-4	12‐core systemic transrectal biopsy	68Ga‐PSMA‐11 PET/CT	SUVmax	RP pathology	RP ISUP GG ≥ biopsy ISUP GG
Xue 2022 (19)	88	Biopsy ISUP GG 2	transperineal template biopsy and MRI‐targeted biopsies	68Ga-PSMA PET/CT	SUVmax ≥ 5.4	RP pathology	RP ISUP GG ≥ 3
Yin 2021 (20)	67	Biopsy ISUP GG 1-4	12-core systematic biopsy and MRI-targeted biopsy	68Ga-PSMA- 11 PET/CT	SUVmax ≥ 12.16	RP pathology	RP ISUP GG ≥ biopsy ISUP GG
Zheng 2023 (21)	89	Biopsy ISUP GG 1-4	12–14 core TURS-guided systematic prostate biopsy	18F-PSMA-1007 PET/CT	PSMA-TL ≥ 95.268	RP pathology	RP ISUP GG ≥ biopsy ISUP GG
Zang 2023 (22)	87	Biopsy ISUP GG 1-4	12-core TURS-guided systematic prostate biopsy	68Ga-PSMA- 11 PET/ CT	deep learning model	RP pathology	RP ISUP GG ≥ biopsy ISUP GG

GG, grade group; ISUP, International Society of Urological Pathology; MRI, magnetic resonance imaging; NA, not applicable; PET/CT, positron emission tomography/computed tomography; PSMA, prostate‐specific membrane antigen; PSMA-TL, total uptake of PSMA-avid lesions; RP, radical prostatectomy; SUVmax, maximum standard uptake value; TURS, transrectal ultrasonography.

**Figure 2:**
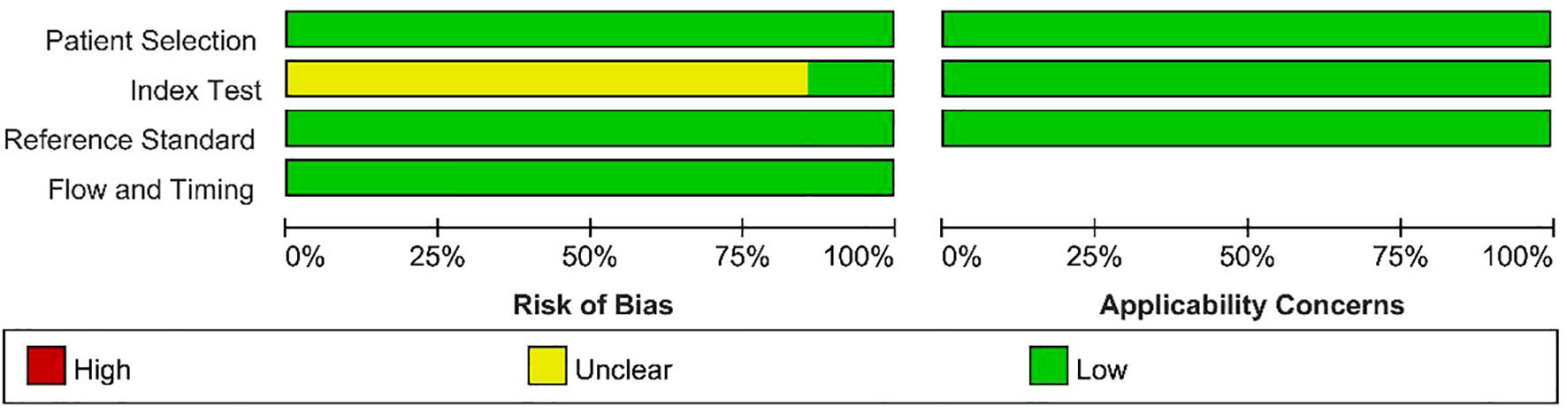
Graph showing the quality assessment of included studies.

**Figure 3:**
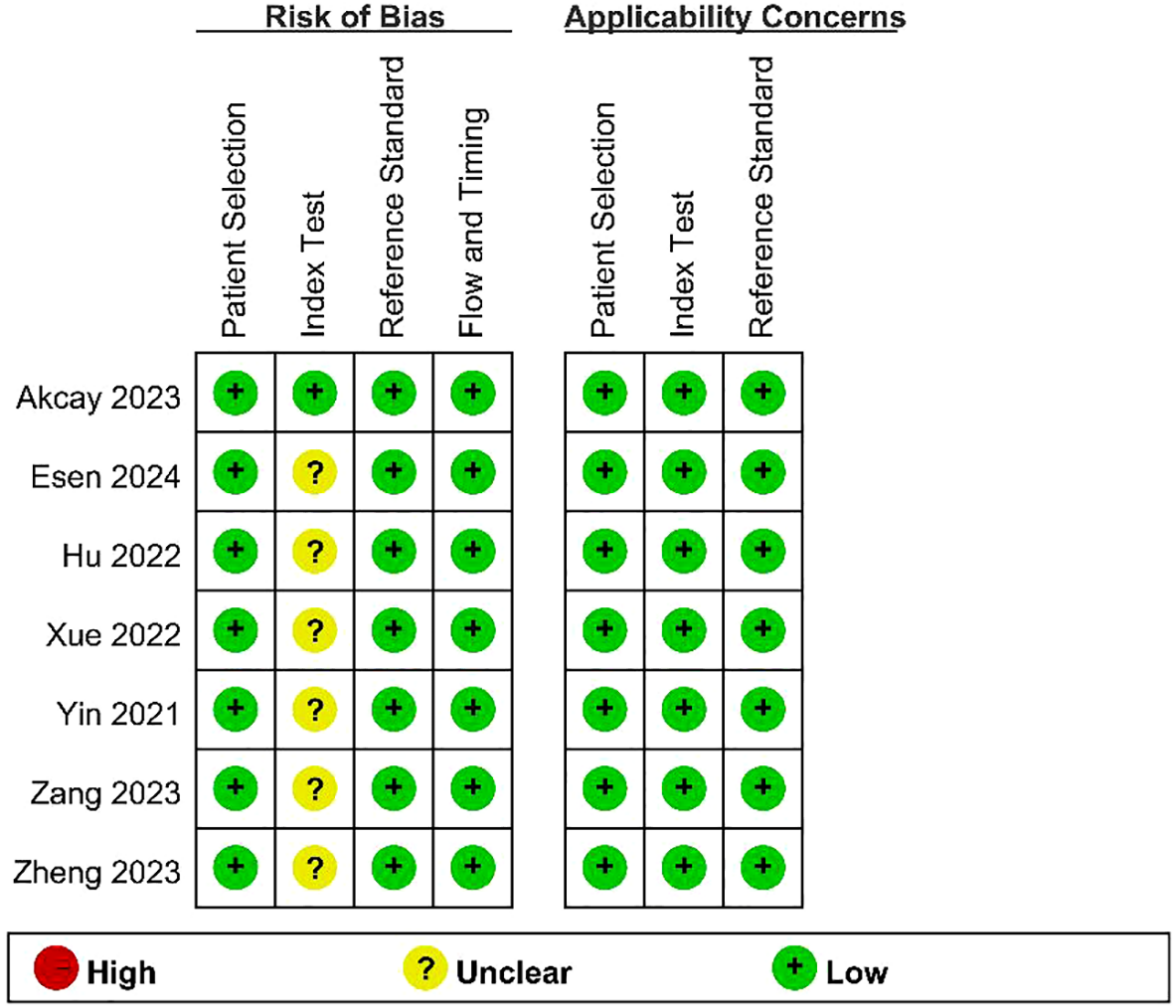
Chart summarizing the quality assessment of included studies.

The results of the quality assessment of the included studies using the QUADAS - 2 tool are presented in **Figure 2** and **Figure 3**. As there was only an uncertain risk of bias, no study was excluded.

### Diagnostic accuracy

**Figure 4:**
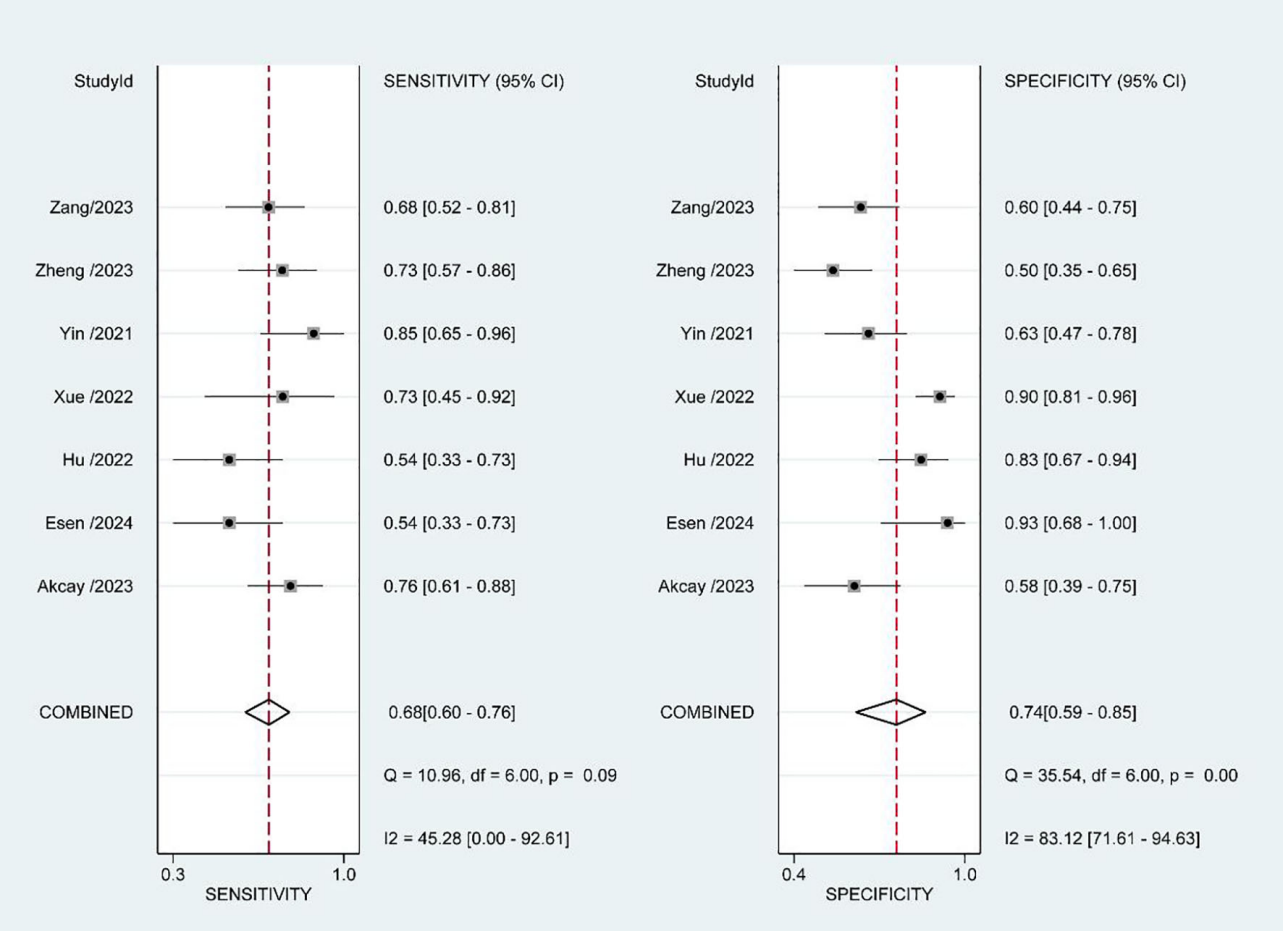
Forest plots of sensitivity and specificity.

**Figure 5:**
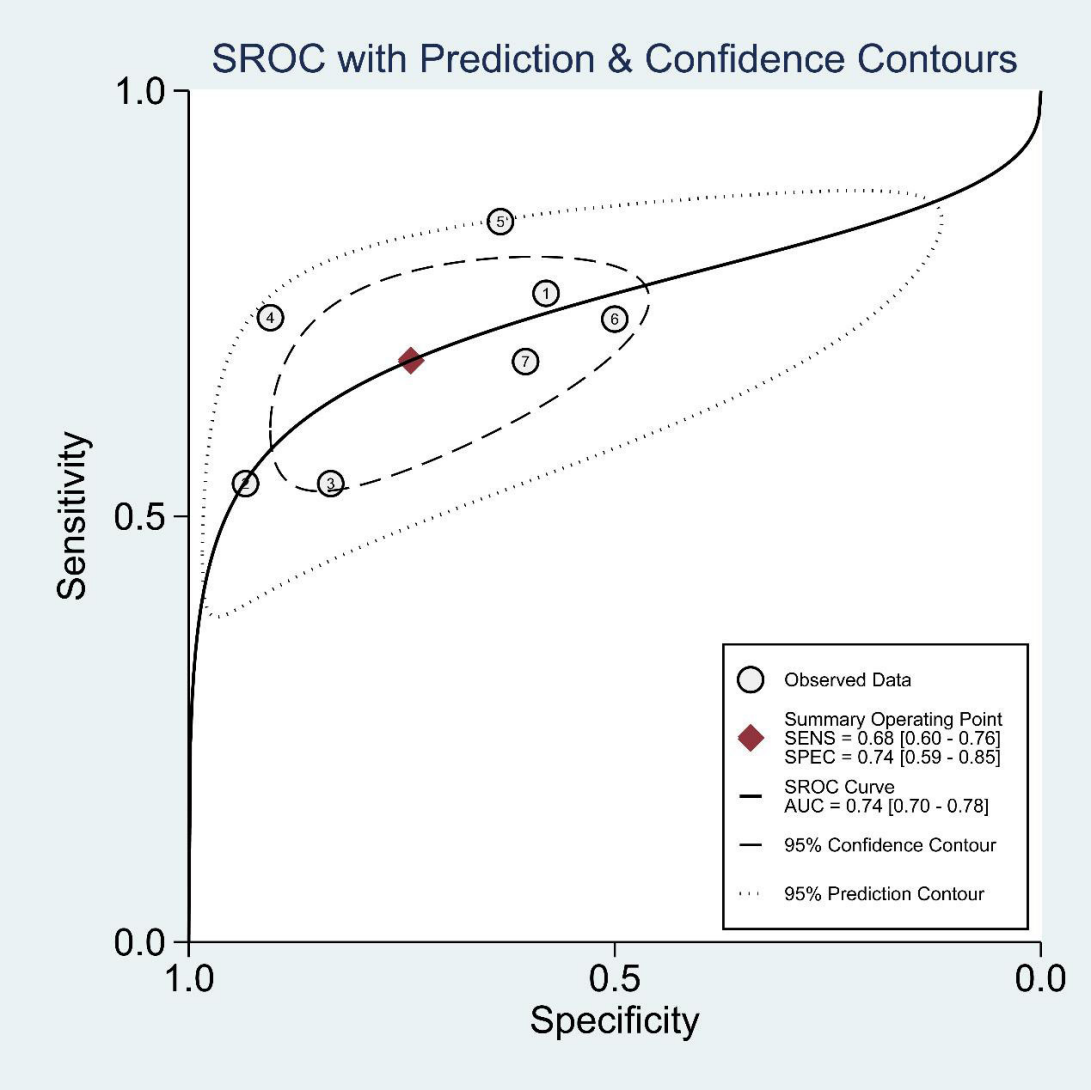
Summary receiver operating characteristic curve.

The estimated pooled sensitivity, specificity, positive predictive value, and negative predictive value for the prediction of pathological upgrading using PSMA-PET/CT are as follows: the sensitivity is 0.68 (95% CI, 0.60 - 0.76), the specificity is 0.74 (95% CI, 0.59 - 0.85), the positive predictive value is 0.68 (95% CI, 0.58 - 0.79), and the negative predictive value is 0.73 (95% CI, 0.61 - 0.86). **Figure 4** presents a forest plot illustrating the sensitivity and specificity values from each study conducted in this analysis. The diagnostic odds ratio was determined to be 6 (95% CI, 3 - 11), with the positive likelihood ratio calculated as 2.6 (95% CI, 1.7 - 4.1). Additionally, a low value of 0.43 (95% CI, 0.34 - 0.53) was obtained for the negative likelihood ratio. An overview of estimated diagnostic accuracy observed in each study can be found in **Table 2**. Moreover, the SROC curve in **Figure 5** demonstrates an area under the curve of 0.74 (95% CI, 0.70 - 0.78).

**Table 2:** Diagnostic estimates of each study.

First author year	Diagnostic estimates (95%CI)								
	True positive	False positive	False negative	True negative	Positive likelihood ratio	Negative likelihood ratio	Diagnostic odds ratio	Sensitivity	Specificity
Akcay 2023 (16)	32	13	10	18	1.82 [1.16 - 2.84]	0.41 [0.22 - 0.76]	4.43 [1.62 - 12.12]	0.76 [0.61 - 0.88]	0.58 [0.39 - 0.75]
Esen 2024 (17)	14	1	12	14	8.08 [1.18 - 55.46]	0.49 [0.32 - 0.77]	16.33 [1.86 -143.10]	0.54 [0.33 - 0.73]	0.93 [0.68 - 1.00]
Hu 2022( 18)	14	6	12	30	3.23 [1.43 - 7.28]	0.55 [0.36 - 0.86]	5.83 [1.82 - 18.75]	0.54 [0.33 - 0.73]	0.83 [0.67 - 0.94]
Xue 2022 (19)	11	7	4	66	7.65 [ 3.55 - 16.48]	0.29 [0.13 - 0.68]	25.93 [6.49 - 103.52]	0.73 [0.45 - 0.92]	0.90 [0.81 - 0.96]
Yin 2021 (20)	22	15	4	26	2.31 [ 1.50 - 3.57]	0.24 [0.10 - 0.62]	9.53 [2.76 - 32.96]	0.85 [0.65 - 0.96]	0.63 [0.47 - 0.78]
Zheng 2023 (21)	30	24	11	24	1.46 [1.04 - 2.05]	0.54 [0.30 - 0.96]	2.73 [1.12 - 6.66]	0.73 [0.57 - 0.86]	0.50 [0.35 - 0.65]
Zang 2023 (22)	30	17	14	26	1.72 [ 1.13 - 2.63]	0.53 [0.32 - 0.86]	3.28 [1.36 - 7.91]	0.68 [0.52 - 0.81]	0.60 [0.44 - 0.75]

CI, confidence interval.

### Evaluation of bias and heterogeneity

We utilized the I^2^ method to evaluate heterogeneity and observed moderate heterogeneity in sensitivity (p=0.09, I^2^ = 45.28), as well as high heterogeneity in specificity (p<0.01, I^2^ = 83.12). We hypothesized that the heterogeneity observed among studies could potentially be attributed to variations in biopsy strategies employed across different studies, as well as disparities in the Gleason scores of patients included in each study. To investigate potential sources of heterogeneity, we performed subgroup analyses by stratifying the study into the following subgroups: (1) based on the biopsy strategy, three studies employed a combined systemic biopsy and MRI-targeted biopsy approach, while the remaining three studies utilized only systemic biopsy; (2) considering patient characteristics, three studies included patients with a biopsy ISUP GG of less than or equal to 2, whereas the other four studies enrolled patients with biopsy ISUP GG ranging from 1 to 4. However, regression analysis results (**Table 3**) indicated that neither the biopsy strategy nor patient inclusion criteria had any impact on heterogeneity. Consequently, the source of heterogeneity remains unclear.

**Table 3:** Meta-regression and subgroup analysis of the included studies.

Variables	Subgroup	Number of studies	Number of patients	Sensitivity, 95% CI	P	Specificity, 95% CI	P
Biopsy ISUP GG	≤ 2	3	202	0.67 [0.54 - 0.80]	0.16	0.84 [0.71 - 0.97]	0.52
	1-4	4	305	0.70 [0.59 - 0.80]		0.65 [0.48 - 0.82	
Biopsy process	Containing MRI-targeted-biopsy	3	196	0.71 [0.55 - 0.86]	0.67	0.85 [0.73 - 0.98]	0.40
	Systematic biopsy only	3	238	0.66 [0.51 - 0.80]		0.66 [0.45 - 0.87]	

CI, confidence interval; GG, grade group; ISUP, International Society of Urological Pathology; MRI, magnetic resonance imaging.

**Figure 6:**
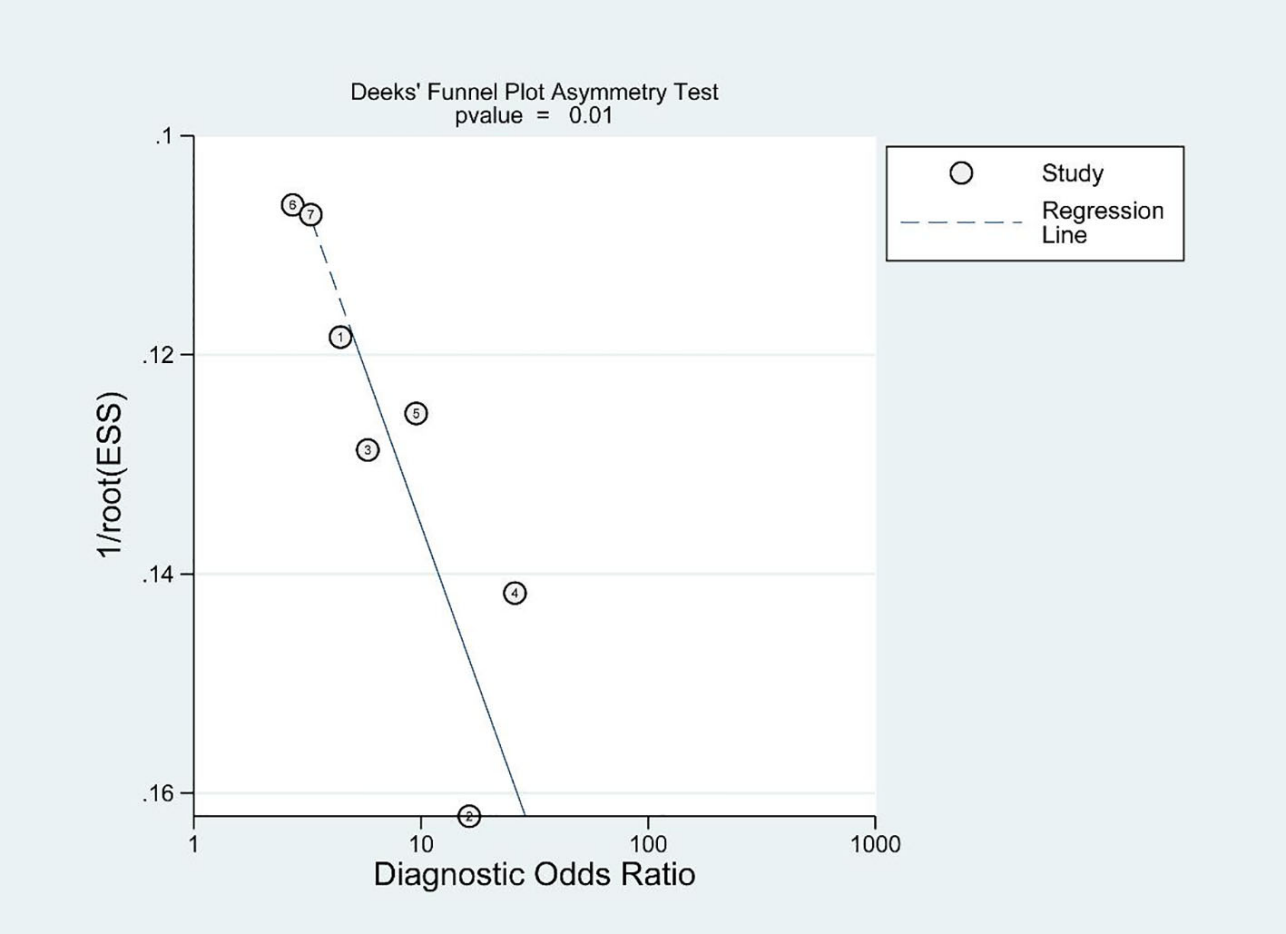
Deeks’ funnel plots of publication bias.

Deeks’ funnel plot demonstrates the presence of publication bias, as indicated by a significant bias coefficient of 39.8 (p<0.05), as depicted in **Figure 6**.

## Discussion

The significant heterogeneity of prostate cancer is widely acknowledged in terms of its biological characteristics (23). Urologists have long been striving to stratify the risk levels of prostate cancer and provide tailored treatments accordingly (24). Another noteworthy aspect of prostate cancer is the potential discordance between biopsy pathology and postoperative pathology, which presents challenges in pre-surgical risk stratification (25). Consequently, there has been a growing research interest in identifying effective tools for predicting postoperative pathology, making it a prominent area of investigation (7, 26, 27). In recent years, PSMA PET/CT has gained increasing popularity in clinical practice. Not only does it exhibit superior diagnostic accuracy compared to traditional imaging techniques in lymph node staging and bone metastasis staging, but it also demonstrates high precision in evaluating local lesions associated with prostate cancer (28). In the field of radiation oncology, PSMA PET/CT is expected to contribute to more accurate target delineation and individualized chemoradiotherapy strategies, thereby advancing the development of precision therapy. Consequently, some scholars have initiated investigations into the utility of PSMA PET/CT as a predictive tool for pathological upgrading after radical prostatectomy (29–31); however, there remains a scarcity of relevant studies on this topic.

The present systematic review and meta-analysis represents the first comprehensive investigation focusing on the predictive value of PSMA PET/CT in relation to pathological upgrading subsequent to radical prostatectomy. The findings suggest that PSMA PET/CT demonstrates a moderate level of accuracy (with an area under the ROC curve of 0.74) when employed for this specific purpose (32).

The present study has identified limited evidence suggesting the promising potential of PSMA PET/CT in predicting pathological upgrading of prostate cancer. However, it is important to note that the available evidence does not yet establish this modality as mature and refined. Firstly, its accuracy remains moderate. It is worth acknowledging that high accuracy cannot be expected from a single method alone, and combining multiple indicators usually yields better results. For instance, Hu et al. integrated PSMA PET/CT with variables including prostate volume, BMI, the number of positive biopsy cores, and other indicators to develop a nomogram tool for prognosticating pathological upgrading in prostate cancer patients, resulting in enhanced predictive accuracy rates (18). We anticipate that further exploration will prompt more scholars to integrate PSMA PET/CT with other clinicopathological indicators in order to enhance prediction accuracy in the future.

The determination of positive results for PSMA PET/CT poses another challenge. The 7 articles included in this review employ different criteria, with 5 articles utilizing SUVmax-related indicators as the standard; however, their cut-off values vary. Akcay et al. consider a PRIMARY score3–5 as indicative of positivity (16). The PRIMARY score is a standardized evaluation system for PSMA PET/CT proposed by the European Society of Nuclear Medicine in 2022, primarily used to assess local lesions of prostate cancer and predict clinically significant cases (33). Utilizing this standardized score to predict pathological upgrades also represents a future research direction. Zang et al. utilize a deep learning model to interpret and determine the outcomes of PSMA PET/CT, which constitutes an important attempt; nevertheless, further relevant research is required (22).

The literature included in this study demonstrated a significant degree of heterogeneity, prompting efforts to identify the underlying sources. Subgroup analysis was conducted based on the biopsy strategy employed and the patients’ biopsy Gleason score. Surprisingly, the results did not indicate that these two factors contributed to the observed heterogeneity, contrary to our initial expectations. Previous research has suggested that patients undergoing MRI-targeted biopsies exhibit a lower rate of postoperative pathological upgrading compared to those undergoing systematic biopsies, potentially introducing variability in the diagnostic accuracy of PSMA-PET/CT for these patient cohorts (34). On the other hand, PSMA-PET/CT exhibits a high predictive value for clinically significant prostate cancer, typically defined as ISUP GG ≥ 2 (35). Therefore, there may be variations in the accuracy of predicting postoperative pathological upgrading for patients with biopsy ISUP GG ≤ 2 and biopsy ISUP GG 1–4 using PSMA-PET/CT. We hoped that subsequent studies can discuss potential strategies to enhance its predictive performance, such as integrating other clinical indicators, advanced imaging techniques, or machine learning models. Our subgroup analysis did not confirm our expectations, possibly due to limitations in study numbers. It is worth pointing out that the seven included studies used different criteria for psma pet positivity, as shown in **Table 1**, and this difference may have led to inconsistencies in diagnostic categorization, which in turn may have affected the combined sensitivity and specificity estimates, and may have been one of the important reasons for the significant heterogeneity in this study. Other potential sources of heterogeneity, such as interpretation and judgment criteria for PSMA PET/CT and types of radiopharmaceutical isotopes used for labeling were not discussed in this paper due to limitations in literature quantity. As more relevant research is conducted in the future, further exploration into sources of heterogeneity will be necessary.

This study is subject to certain limitations. Firstly, the source of heterogeneity remains unclear due to the limited number of studies included in this meta-analysis, which hinders our ability to conduct subgroup analysis on potential confounding factors. The limited number of studies also prevented us to analyze the AUC, accuracy (ACC), sensitivity, specificity, advantages and disadvantages of 68 Ga-PSMA-PET/CT and 18F-PSMA-PET/CT separately in this study. We lacked specific information, due to limitations such as insufficient data, regarding the intervention thresholds. Therefore, the Decision Curve Analysis (DCA) was not used in this study. We recommend conducting more prospective studies to address the limitations arising from insufficient data, to better characterize the performance of PSMA PET/CT across diverse patient populations, to further investigate performance differences between imaging modalities, and to incorporate Decision Curve Analysis (DCA) in future research. Secondly, the varying criteria for PSMA-PET/CT positivity among the included studies, five of which used SUVmax as a criterion for determination, however, the specific thresholds were inconsistent; another study used the PRIMARY scoring system, the Another study used the PRIMARY scoring system, and another used the DEEP LEARNING model for the determination. Different Positive test criteria may reduce the stability and interpretability of the combined analysis and pose a challenge for applying the results of this study to actual clinical scenarios. We endorse Akcay’s study, which employed a standardized PRIMARY score for evaluating and determining PSMA-PET/CT positivity. Standardization of the evaluation system is crucial for enhancing its clinical applicability. Thirdly, the population included in this study consisted of patients with ISUP GG 1-4; therefore, the findings of this study may not be applicable to active surveillance patients. It is recommended that future studies include a larger proportion of low-risk prostate cancer patients as predicting pathological upgrade in this subgroup can significantly impact treatment decisions. Fourth, the incidence of pathological upgrade is undoubtedly influenced by the biopsy strategy, and there was inconsistency in the biopsy strategies employed across the studies included in this meta-analysis. A few studies utilized MRI-targeted biopsies, while none attempted PSMA-PET/CT-targeted biopsies, which could potentially impact our findings. Fifth, our results indicate the presence of publication bias and it should be noted that only English literature was included in this analysis, which may introduce a potential source of bias. Sixth, the data analyzed in this study were limited to PSMA PET/CT and did not involve data related to mpMRI. Therefore, we were unable to perform a direct comparative analysis. We suggest that future studies continue to compare the performance of PSMA PET/CT and mpMRI.

## Conclusions

The diagnostic accuracy of PSMA-PET/CT in predicting pathological upgrading after radical prostatectomy in patients with prostate cancer is moderate; however, despite its imperfections, this method demonstrates significant potential for application and warrants further research in related fields.

## Data Availability

The original contributions presented in the study are included in the article/supplementary material. Further inquiries can be directed to the corresponding author.
